# Fibroblast Growth Factor 2 Attenuates Renal Ischemia-Reperfusion Injury via Inhibition of Endoplasmic Reticulum Stress

**DOI:** 10.3389/fcell.2020.00147

**Published:** 2020-03-24

**Authors:** Xiaohua Tan, Qianyu Tao, Guixiu Li, Lijun Xiang, Xiaomeng Zheng, Tianzhen Zhang, Cuijiao Wu, Dequan Li

**Affiliations:** ^1^Department of Pathology, School of Basic Medicine, Qingdao University, Qingdao, China; ^2^School of Pharmaceutical Sciences, Wenzhou Medical University, Wenzhou, China; ^3^Beilun District People’s Hospital of Ningbo, Ningbo, China; ^4^Outpatient Operating Room, Jiaozhou Central Hospital of Qingdao, Qingdao, China; ^5^Department of Human Anatomy, Histology and Embryology, School of Basic Medicine, Qingdao University, Qingdao, China; ^6^Department of Traumatology Medicine, The First Affiliated Hospital of Wenzhou Medical University, Wenzhou, China

**Keywords:** ischemia-reperfusion, acute kidney injury, FGF2, apoptosis, ER stress

## Abstract

Acute kidney injury (AKI) is a serious clinical disease that is mainly caused by renal ischemia-reperfusion (I/R) injury, sepsis, and nephrotoxic drugs. The pathologic mechanism of AKI is very complex and may involve oxidative stress, inflammatory response, autophagy, apoptosis, and endoplasmic reticulum (ER) stress. The basic fibroblast growth factor (FGF2) is a canonic member of the FGF family that plays a crucial role in various cellular processes, including organ development, wound healing, and tissue regeneration. However, few studies have reported the potential therapeutic effect of FGF2 in the repair of renal ischemic injury in the past two decades. In the present study, we investigated the protective effect of FGF2 on renal I/R injury using Sprague-Dawley and NRK-52E cells. Our results showed that FGF2 significantly attenuates the apoptosis of kidney tissues after I/R injury through the inhibition of excessive ER stress. Moreover, FGF2 also alleviated the excessive ER stress and apoptosis in cultured NRK-52E cells injured by tert-Butyl hydroperoxide (TBHP). Significantly, phosphatidylinositol 3-kinase (PI3K)-selective inhibitor LY294002 and mitogen-activated protein kinase kinase (MEK)-selective inhibitor U0126 were utilized in the present study to examine the protective mechanism of FGF2. Our *in vitro* experimental results confirmed that both LY294002 and U0126 largely abolished the protective effect of FGF2. Taken together, the findings of the present study indicated that FGF2 attenuates I/R-induced renal epithelial apoptosis by suppressing excessive ER stress via the activation of the PI3K/AKT and MEK-ERK1/2 signaling pathways.

## Introduction

Despite the advances in medical technology in the past decades, acute kidney injury (AKI) remains a serious medical problem associated with high morbidity and mortality (Ishani et al., 2009). Ischemia-reperfusion (I/R) injury, which can be elicited in many conditions including surgery, hemorrhagic shock, and kidney transplantation, is the primary cause of AKI (Eltzschig and Eckle, 2011; Basile et al., 2012). Currently, many clinical and experimental studies have investigated renal I/R injury. However, the pathogenesis of AKI caused by I/R injury is not yet fully understood (Chertow et al., 2005; Wu et al., 2018). The pathologic mechanism of AKI is complicated and may involve inflammation, microvascular dysfunction, autophagy, apoptosis, oxidative stress, mitochondrial damage, and maladaptive endoplasmic reticulum (ER) stress (Bonventre and Yang, 2011). Extensive renal tubular cell death, including apoptosis and necrosis, leading to interstitial edema and decline of glomerular filtration rate (GFR), is the critical factor in the occurrence of AKI (Bohle et al., 1976; Winterberg and Lu, 2012). So far, there are no reliable drugs or surgical therapies for the prevention and treatment of AKI (Soares et al., 2019). Effective treatment for AKI is urgently needed.

Endoplasmic reticulum plays an important role in protein homeostasis and is extremely sensitive to changes in the cellular microenvironment (Brodsky and Skach, 2011; Walter and Ron, 2011; Cybulsky, 2017). Under various pathologic conditions, increased demand for protein folding or disruption of normal protein folding lead to excessive accumulation of unfolded or misfolded proteins in ER and thus elicit ER stress and the unfolded protein response (UPR). Currently, the role of ER stress in the regulation of cell growth, cell migration, cell differentiation, and apoptosis has been confirmed. Many studies have shown the relationship between excessive activation of ER stress and various kidney diseases, including AKI, chronic kidney disease (CKD), glomerular disease, and diabetic nephropathy (Inoue et al., 2019). Apoptosis, a form of programed cell death characterized by DNA cleavage and nuclear condensation, has been observed and well recognized in AKI (Schumer et al., 1992; Linkermann et al., 2014). Many studies have reported the significant role of ER stress in the process of apoptosis (Hetz, 2012; Wang et al., 2012). Therefore, a therapeutic strategy aimed at the inhibition of tubular cell apoptosis induced by ER stress may facilitate the treatment of AKI after renal I/R injury.

The basic fibroblast growth factor (FGF2) is a canonic member of the FGF family, which mediates various cellular processes through binding and activating relevant FGF receptors (FGFR) (Li et al., 2016). The therapeutic potential of FGF2 for cardiovascular disease, wound healing, and tissue regeneration has been identified (Beenken and Mohammadi, 2009; Xiao et al., 2010). Furthermore, previous studies revealed that FGF2 expression is upregulated in kidney during the recovery phase after I/R injury (Villanueva et al., 2006). Our previous study also verified the regulation of FGF2 in oxidative stress and inflammatory response after renal I/R injury (Tan et al., 2017). As mentioned above, ER stress plays a crucial role in the apoptosis of renal tubular epithelial cells after ischemic injury. However, the relationship between the protective effect of FGF2 and ER stress during AKI has not yet been completely clarified. The mitogen-activated protein kinases (MAPKs), a type of serine/threonine kinase, are involved in the regulation of various cellular processes, such as cell survival, proliferation, differentiation, and apoptosis (Kwon et al., 2006). The extracellular signal-regulated kinase (ERK) signaling pathway is an important member of MAPK, which is activated by MAPK/ERK kinase (MEK) in response to multiple stimuli, including growth factors, pro-inflammatory cytokines, and viral infection (Pearson et al., 2001). Many studies have reported that renal tubular epithelial cell survival after I/R injury is dependent on the activation of the ERK pathway (di Mari et al., 1999; Kwon et al., 2006). Protein kinase B (PKB), also known as Akt, is also involved in the repair process during renal I/R injury (Cai and Semenza, 2004). The relationship between the activation of the PI3K/Akt and MEK-ERK1/2 pathways and the regulation of ER stress has been studied in various diseases (Wang et al., 2012; Hsu et al., 2017). However, it is not clear whether the activation of PI3K/Akt and MEK-ERK1/2 pathways is involved in the protective effect of FGF2 after reperfusion.

In the present study, we hypothesized that FGF2 can protect kidneys from I/R injury by inhibiting renal tubular epithelial cell apoptosis and suppressing excessive ER stress. The role of FGF2 in the regulation of apoptosis and ER stress depends on the activation of PI3K/Akt and MEK-ERK1/2 signaling pathways. We verified the hypothesis with a kidney ischemia-reperfusion injury model in SD rat and NRK-52E cells injured by TBHP. Our results confirmed that the protective effect of FGF2 against I/R injury in kidney is intimately related to ER stress, which is, at least partially, mediated by the PI3K/Akt and MEK-ERK1/2 signaling pathways.

## Materials and Methods

### Reagents and Antibodies

Recombinant human FGF2, Tert-Butyl hydroperoxide (TBHP) solution, LY294002 (selective PI3K inhibitor), and U0126 (selective MKK1/2 inhibitor) were purchased from Sigma-Aldrich Corp. (St. Louis, MO, United States). Fetal bovine serum (FBS), Dulbecco’s Modified Eagle Medium (DMEM) and 4′, 6-Diamidino-2-phenylindole (DAPI) were purchased from Invitrogen/Gibco Corp. (Carlsbad, CA, United States). Antibodies against cleaved Caspase-3 (catalog number: 9661), Akt (catalog number: 4685), phospho-Akt (catalog number: 4060), and phospho-ERK1/2 (catalog number: 9101) were purchased from Cell Signaling Technology (CST) Inc. Anti-ERK1/2 antibody (catalog number: 82380) was purchased from Thermo Fisher Scientific (Sunnyvale, CA, United States). Antibodies against CHOP (catalog number: ab11419), GRP78 (catalog number: ab21685), ATF-6 (catalog number: ab203119), XBP1 (catalog number: ab37152), β-actin (catalog number: ab179467), and GAPDH (catalog number: ab9485) were purchased from Abcam Inc. (Cambridge, MA, United States). The secondary antibodies were purchased from Cell Signaling Technology (CST) Inc. (Danvers, MA, United States) or Santa Cruz Biotechnology (Santa Cruz, CA, United States). The terminal deoxynucleotidyl transferase-mediated dUTP nick-end labeling (TUNEL) Assay Kit was purchased from Abcam, Inc. (Cambridge, MA, United States).

### Renal I/R Injury Model and Assessment of Renal Function

A renal I/R injury model was established in rats to clarify the protective effect of FGF2 against I/R injury in kidney. Male Sprague-Dawley (SD) rats (8 weeks old) were purchased from Beijing Vital River Laboratory Animal Technology Co., Ltd., and were housed in an Specific-pathogen-free (SPF) facility. The experimental protocol in the present study was approved by the Institutional Animal Ethical and Use Committee of Wenzhou Medical University. The renal I/R injury model was created as we described in previous studies (Tan et al., 2013, 2017). Briefly, SD rats were anesthetized with an intraperitoneal (ip) injection of 25 mg/kg of Pentobarbital sodium and placed on a thermostatic surgical pad. The right kidney was carefully liberated from surrounding tissue, and nephrectomy was performed. The left kidney was exposed after a midline incision, and the renal artery was occluded with a vascular clamp for 45 min, after which renal blood flow was re-established. For measurement of renal function, serum creatinine (Cr) was measured at 6, 24, and 72 h after renal ischemia-reperfusion in the hospital medicine biochemical laboratory (the First Affiliated Hospital of Wenzhou Medical University). Kidneys were harvested and stored in a cryogenic refrigerator for further experiments. Rats were randomly divided into four groups in the present study. (a) Sham group: 0.5 ml saline was given to rats by ip injection half an hour before sham operation; (b) FGF2 group: 0.5 mg/kg FGF2, dissolved in equivalent amount of sterile saline, was given to rats by ip half an hour before sham operation; (c) I/R group: kidneys were subjected to 45 min of ischemia through clipped renal artery followed by reperfusion; (d) I/R-FGF2 group: 0.5 mg/kg FGF2 was given to rats by ip half an hour before renal ischemia operation.

### Cell Culture

Cell culture was performed to further clarify the role of the PI3K/AKT and MEK-ERK1/2 signaling pathways in the protective effect of FGF2. NRK-52E, a rat renal tubular epithelial cell line, was used. The NRK-52E cell line was purchased from the American Type Culture Collection (Manassas, VA, United States) and maintained in DMEM supplemented with 10% FBS and incubated under 37°C, 95% air, and 5% CO_2_. NRK52E cells were plated at 2 × 10^5^ in each 35-mm culture dish for 24 h, and then serum starvation was performed for 12 h in DMEM containing 1% FBS. Serum stimulation was performed by switching the culture medium to 10% FBS after serum starvation. TBHP is an oxidant for I/R injury in various cultured cells, including renal epithelial cells (Paller and Manivel, 1992; Wu et al., 2017; Chen et al., 2018; Shen et al., 2019). In order to determine the effect of FGF2 on TBHP-induced ER stress, NRK-52E cells were divided into six groups randomly: (a) Vehicle group: NRK-52E cells were cultured in complete medium without any supplement; (b) FGF2 group: NRK-52E cells were cultured in complete medium and treated with recombinant human FGF2 (100 ng/ml) for 2 h; (c) TBHP group: NRK-52E cells were cultured in complete medium, and then TBHP (200 μmol/L) was added for an additional 12 h; (d) TBHP-FGF2 group: NRK-52E cells were pretreated with recombinant human FGF2 (100 ng/ml) for 2 h, and then TBHP (200 μmol/L) was added for an additional 12 h; (e) LY294002 group: NRK-52E cells were pretreated for 2 h with specific Akt pathway inhibitor LY294002 (20 μmol/L), and then cells were treated as in the TBHP + FGF2 group. (f) U0126 group: NRK-52E cells were pretreated for 2 h with specific ERK1/2 pathway inhibitor U0126 (20 μmol/L), and then cells were treated as in the TBHP-FGF2 group. All experiments were repeated three times.

### Western Blot Analysis

The regulation role of FGF2 on ER stress and apoptosis was assessed by analyzing the expression of proteins by Western blot. For protein analysis of renal tissue in different groups, kidney tissues (containing both cortex and medulla but not containing renal fibrous capsule) were homogenized, and total proteins were extracted using tissue lysis buffer. For protein analysis of *in vitro* samples, NRK-52E cultured in a petri dish was rinsed with PBS buffer three times, and total proteins were extracted using cell lysis buffer. An equivalent of 100 μg protein extracted from kidney tissues (or 30 μg protein extracted from NRK-52E cells) was separated by SDS-PAGE gels and then transferred onto PVDF membrane for immunoblot analysis with the primary antibodies directed against the relevant proteins. Primary antibodies against cleaved Caspase-3 (1:1000), Bax (1:3000), Bcl-2 (1:1000), GRP78 (1:1000), CHOP (1:5000), XBP-1 (1:1000), ATF-6 (1:2000), Akt (1:1000), phosphor-Akt (1:1000), ERK1/2 (1:1000), and phosphor-ERK1/2 (1:1000) were used in the present study. GAPDH (1:2500) or β-actin (1:2000) were used to quantify the protein expression levels. The signals were visualized with the ChemiDoc^TM^ XRS + Imaging System (Bio-Rad Laboratories). The band densities were quantified with Multi Gauge Software of Science Lab 2006 (FUJIFILM Corporation, Tokyo, Japan).

### Renal Immunofluorescence and Immunohistochemistry

Immunohistochemistry and immunofluorescence staining were performed in order to observe the expression and location of ER stress- and apoptosis-relevant proteins in kidney. Kidneys were excised and harvested 6, 24, or 72 h following I/R injury. Renal tissue embedded in paraffin was cut into 5-μm sections and then incubated with antibodies against cleaved Capase-3, GRP78, and CHOP at 4°C overnight. After being incubated with primary antibodies, the slides were then incubated with secondary antibodies for 1 h at room temperature. Cell apoptosis in the kidney was detected using the terminal deoxynucleotidyl transferase-mediated dUTP nick-end labeling (TUNEL) assay kit (Roche, Mannheim, Germany). The apoptosis index was analyzed based on five randomly selected images from each group. All images were captured under a Laser confocal microscope (Nikon, Ti-E&A1 plus).

### Renal Histopathology Damage Assessment

In order to evaluate the degree of renal histopathology damage, sections were stained with hematoxylin and eosin (H&E). Images were captured under a light microscope (Nikon ECLIPSE 80i). The degree of renal histopathology damage was evaluated based on intraluminal necrotic cells, cell swelling, interstitial congestion, edema, and protein casts. The following 5-point scoring system was utilized to assess renal damage: 0 points - normal renal morphology, 1 point - damage of kidney tissue ≤ 10%, 2 points - damage of kidney tissue 11–25%, 3 points - damage of kidney tissue 26–45%, 4 points - damage of kidney tissue 46–75%, and 5 points - damage of kidney tissue ≥ 76%. The pathologists assessing the images were blinded to allocation group.

### Statistical Analysis

The statistical evaluation of the data was performed using one-way Analysis of Variance (ANOVA), and when more than two groups were compared, the statistical evaluation of the data was performed using two-way ANOVA. Values of ^∗^*P* < 0.05, *^∗∗^P* < 0.01, and *^∗∗∗^P* < 0.001 were considered statistically significant.

## Results

### FGF2 Ameliorates Renal Histological Damage and Protects Renal Function via the Activation of FGFR After I/R Injury

**FIGURE 1:**
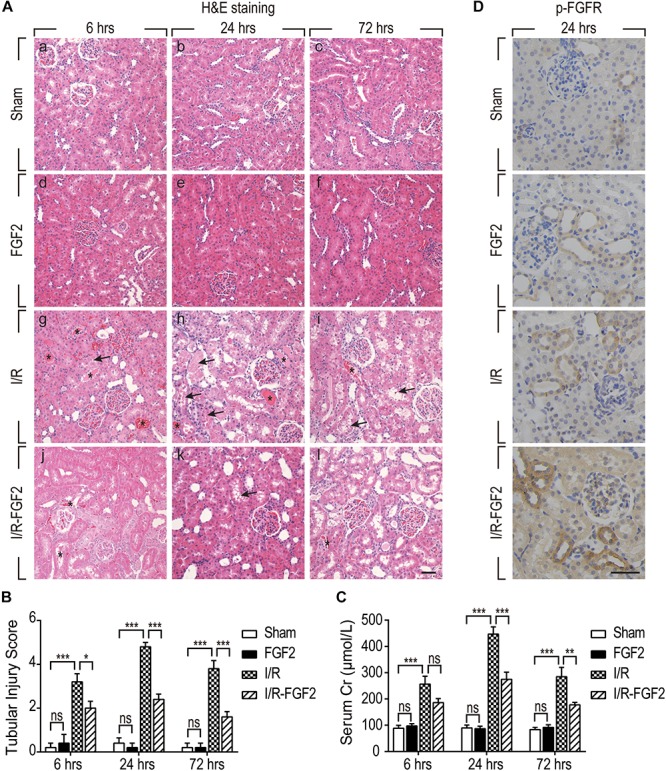
Fibroblast growth factor 2 protects kidney against renal I/R injury via the activation of FGFR. **(A)** Renal histological evaluations were assessed with H&E staining at 6, 24, and 72 h after reperfusion. Asterisks show interstitial congestion, edema, and proteinaceous casts. Arrows represent intraluminal necrotic cellular debris. Images are representative of five animals in each group. Scale bar represents 50 μm. **(B)** Renal tubular injury scores were quantified and analyzed based on H&E staining. Results are representative of five animals in each group. **(C)** Serum creatinine levels of rats from the Sham, FGF2, I/R, and I/R-FGF2 groups at 6, 24, and 72 h after reperfusion. Representative data of five individual samples in each group. **(D)** Immunohistochemistry staining for phosphor-FGFR at 24 h after reperfusion. Images are representative of five animals in each group. Scale bar represents 50 μm. *P < 0.05, **P < 0.01, ***P< 0.001; *ns* indicates not statistically significant.

In the present study, a rat model of renal I/R injury was established to assess the protective effect of FGF2 on AKI. As shown in Figure 1A, renal histomorphology changes were assessed by H&E staining at 6, 24, and 72 h after reperfusion. No obvious renal pathological damage was observed in the kidney tissues of either the Sham group or the FGF2 group (Figure 1A a–f), whereas kidneys of the I/R group rapidly exhibited typical features of AKI (Figure 1A g–i). Renal interstitial congestion proteinaceous casts and edema (shown as asterisks) were observed at 6, 24, and 72 h after reperfusion, and large masses of necrotic cellular debris (shown as arrows) were detected, especially at 24 h after reperfusion. The extent of renal damage of I/R rats at 72 h was reduced compared to at 24 h, which indicates self-recovery of kidney after I/R injury. As shown in Figure 1A j–l, administration of FGF2 significantly attenuated the degree of renal damage and largely preserved renal integrity. As shown in Figure 1B, the degree of tubular injury was quantified based on H&E staining. FGF2 treatment significantly ameliorated kidney damage after I/R injury. There is no significant difference in the renal tubular injury between the I/R-FGF2 group and the Sham group. Serum creatinine (Cr) levels were separately measured at 6, 24, and 72 h after reperfusion to assess renal function. As expected, the level of serum Cr was markedly elevated in the I/R group compared to the Sham group, whereas FGF2 significantly reduced the level of serum Cr in I/R-FGF2 rats compared to that of I/R rats at 24 h and 72 h after reperfusion (Figure 1C).

We further examined the activation of FGFR by immunohistochemistry staining with phosphor-FGFR antibody. FGF family members perform their diverse biological functions through binding and activating FGFR (Beenken and Mohammadi, 2009). As shown in Figure 1D, few phospho-FGFR-weakly positive tubular epithelial cells were detected in kidney tissues of the Sham group. The number of phospho-FGFR-positive epithelial cells was increased in the kidney tissues of the FGF2 group and I/R group. Significantly, the phosphorylation of FGFR was substantially increased in the kidney tissue of the I/R-FGFR group compared to the I/R group. Activation of FGFR in the present study was similar to that in our previous study (Tan et al., 2017). Furthermore, we observed that most of the phospho-FGFR-positive epithelial cells were present in distal tubule. Based on these results, we speculated that FGF2 is responsible for the protective effect on renal I/R injury via the activation of FGFR in distal tubular epithelial cells.

### FGF2 Prevents I/R-Induced Apoptosis of Renal Tubular Cells

**FIGURE 2:**
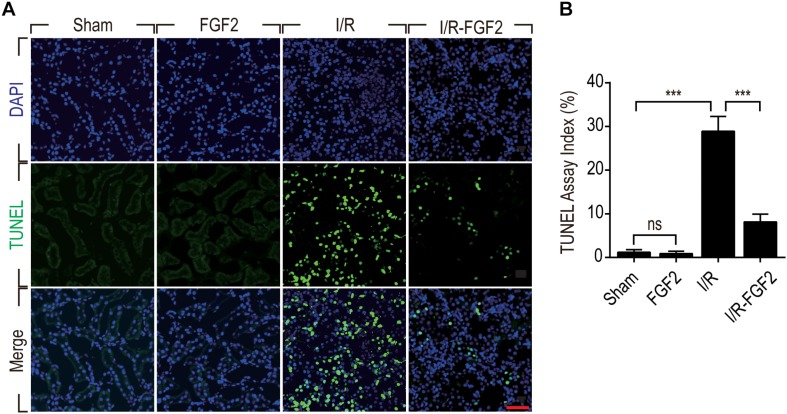
Fibroblast growth factor 2 inhibits cell death in kidneys caused by I/R injury. **(A)** Representative sections of kidney tissues 24 h after reperfusion for the detection of nuclear DNA fragmentation performed by terminal deoxynucleotidyltransferase-mediated dUTP nick-end labeling (TUNEL) staining. Images are representative of five animals in each group. Scale bar represents 50 μm. **(B)** Quantitative analysis of the number of TUNEL-positive renal tubular cells in kidney of each group: 1.18% in the Sham group, 0.89% in the FGF2 group, 28.89% in the I/R group, and 8.15% in the I/R-FGF2 group. FGF2 treatment dramatically decreases the proportion of TUNEL-positive cells in kidney tissues 24 h after reperfusion. Representative data of five individual samples in each group. ***P < 0.001; *ns* indicates not statistically significant.

On the basis of observation of renal histological damage and detection of serum Cr at different time points after reperfusion, we generally find that the extent of renal damage peaked at 24 h after reperfusion. Therefore, we used TUNEL staining to assess apoptosis and necrosis of renal cells 24 h after reperfusion in kidney tissues of each group. As shown in Figure 2A, few TUNEL-positive cells were observed in the kidney tissues of the Sham group and FGF2 group, whereas the proportion of TUNEL-positive cells was dramatically increased in I/R rats. Notably, the proportion of TUNEL-positive cells was much lower in the I/R-FGF2 group compared to the I/R group. Quantification of the proportion of TUNEL-positive cells in kidney revealed that the average percentage of apoptotic cells was 1.18% in the Sham group, 0.89% in the FGF2 group, 28.89% in the I/R group, and 8.15% in the I/R-FGF2 group (Figure 2B). FGF2 treatment dramatically decreased the proportion of TUNEL-positive cells in kidney tissues 24 h after reperfusion.

**FIGURE 3:**
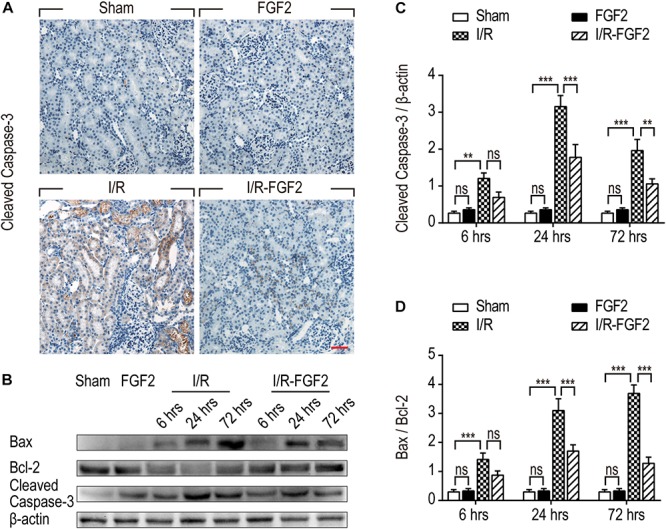
Fibroblast growth factor 2 reduces the expression of pro-apoptotic proteins in kidney tissues. **(A)** Immunohistochemistry staining of cleaved Caspase-3 in kidney tissues at 24 h after reperfusion. The expression of cleaved Caspase-3 was detected in the cytoplasm of renal tubular cells in kidney tissues of the IR group. FGF2 treatment significantly attenuated the expression of cleaved Caspase-3. Images are representative of five animals in each group. Scale bar represents 50 μm. **(B)** Western blotting analysis of expression of cleaved Caspase-3, Bax, and Bcl-2. Total kidney tissues including both cortex and medulla were used to analyze the expression of pro-apoptotic proteins in each group. μ -actinwas used as a loading control. **(C,D)** The bar charts show the normalized optical density analysis. FGF2 significantly reduced the expression of cleaved Caspase-3 and Bax compared with the I/R group. Representative data of five individual samples in each group. **P< 0.01, ***P< 0.001; *ns* indicates not statistically significant.

As TUNEL staining can be used in the identification of both apoptosis and necrosis, expression of key apoptotic proteins, including cleaved Caspase-3, Bax, and Bcl-2, were also detected to distinguish the protective effect of FGF2 against I/R injury. Caspase-3, also known as CPP32, is synthesized as an inactive proenzyme that is processed to the active form (cleaved Caspase-3) in cells undergoing apoptosis (Fernandes-Alnemri et al., 1994). In the present study, increased production of cleaved Caspase-3 was observed in renal tubular epithelial cells of kidney tissues in I/R rats on the basis of immunohistochemistry staining, whereas the expression of cleaved Caspase-3 was strikingly decreased in kidney tissues of the I/R-FGF2 group (Figure 3A). We further used Western blotting to quantify the expression of cleaved Caspase-3, Bax, and Bcl-2 at 6, 24, and 72 h after reperfusion (Figures 3B–D). The production of Bax and cleaved Caspase-3 was markedly increased after I/R injury, whereas the expression of Bcl-2 was decreased. FGF2 can substantially inhibit the expression/activation of Bax and cleaved Caspase-3. Together, the present results indicate that FGF2 administration protects kidneys by preventing apoptosis of renal tubular epithelial cells after I/R injury.

### The Protective Effect of FGF2 on I/R Rats Is Mediated by Inhibition of Excessive ER Stress via the PI3K/AKT and MEK-ERK1/2 Pathways

**FIGURE 4:**
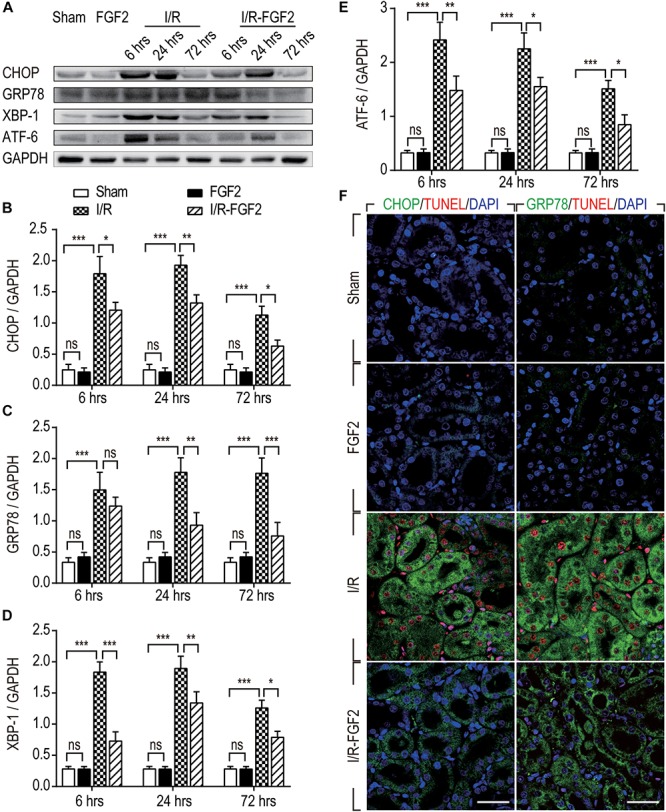
The expression of ER stress-relevant proteins in kidney tissues after reperfusion. **(A)** The expressions of CHOP, GRP78, ATF-6, and XBP-1 were analyzed with Western blotting. GAPDH was used as a loading control. Images are representative of five animals in each group. **(B–E)** The bar charts show the normalized optical density analysis. FGF2 treatment strikingly reduced the expression of CHOP, GRP78, XBP-1, and ATF-6. Representative data of five individual samples in each group. **(F)** Double-staining of CHOP and TUNEL, GRP78 and TUNEL, was detected by immunofluorescence. Images are representative of five animals in each group. *P < 0.05, **P < 0.01, ***P< 0.001; *ns* indicates not statistically significant.

Endoplasmic reticulum stress is generally considered to be an important pathological mechanism in cell survival in various diseases. To investigate the association between the renoprotective effect of FGF2 and ER stress, we examined the expression of ER stress-relevant proteins CHOP, GRP78, XBP-1, and ATF-6, which could participate in the regulation of the unfolded protein response and thus contribute to cellular homeostasis in kidney. As shown in Figures 4A–E, the expression of ER stress-relevant proteins in kidney tissues of I/R rats was dramatically increased, whereas FGF2 treatment significantly reduced their expression. To further investigate the relationship between the protective effect of FGF2 against apoptosis and ER stress, immunofluorescence double-staining was performed at 24 h after reperfusion. As shown in Figure 4F, most TUNEL-positive cells simultaneously exhibit a high expression of CHOP. The results of GRP78 and TUNEL double-staining is similar to the result of CHOP. These results suggest that the administration of FGF2 can effectively attenuate tubular cell apoptosis through inhibition of excessive ER stress induced by I/R injury.

**FIGURE 5:**
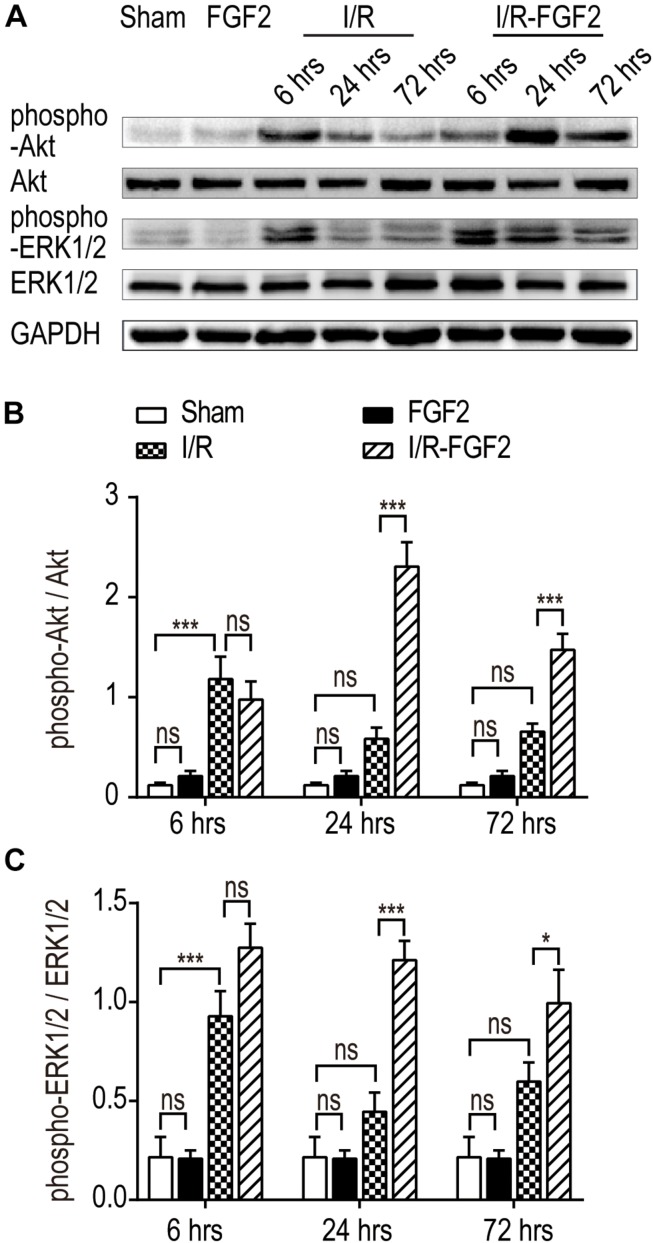
The effect of FGF2 on the phosphorylation of Akt and ERK1/2. **(A)** The phosphorylation of Akt and ERK1/2 was determined by immunoblot analysis. GAPDH was used as a loading control. Images are representative of five animals in each group. **(B,C)** The histograms show the normalized optical density analysis. The phosphorylation of Akt and ERK1/2 in the I/R group was transiently increased at 6 h after reperfusion compared to the Sham group, whereas there was no significant difference between the I/R group and the Sham group at 24 and 72 h. FGF2 treatment strikingly increased the phosphorylation of Akt and ERK1/2 at 24 and 72 h after reperfusion compared to the I/R group. Representative data of five individual samples in each group. *P < 0.05, **P < 0.01, ***P< 0.001; *ns* indicates not statistically significant.

Several studies have revealed the role of ERK1/2 signaling pathways in the resistance of apoptosis via down-regulation of ER stress (Guo et al., 2012; Wang et al., 2012; Bright et al., 2018; Kim and Woo, 2018). Given that FGF2 elicits the regulatory functions by high-affinity binding to FGF receptors and activating downstream signal pathways, we examined the phosphorylation of Akt and ERK1/2 in kidney tissues after I/R injury. As shown in Figures 5A–C, I/R injury caused a transient increase in the phosphorylation of Akt and ERK1/2 compared to the Sham group at 6 h after reperfusion, which indicates the activation of the PI3K/Akt and MEK-ERK1/2 signaling pathways in the self-recovery of kidney tissues after reperfusion. There was no significant difference between the I/R group and the Sham group at 24 and 72 h. FGF2 administration substantially improved the phosphorylation of Akt and ERK1/2 compared to the I/R group at 24 and 72 h after reperfusion. However, there is no significant difference in the activation of the Akt and ERK1/2 pathways between the Sham group and the FGF2 group. Based on these results, we may preliminarily conclude that the role of FGF2 in the regulation of ER stress after renal I/R injury is associated with the activation of the PI3K/Akt and ERK1/2 signaling pathways.

### Inhibition of the PI3K/AKT or MEK-ERK1/2 Pathway Largely Reversed the Protective Effect of FGF2 *in vitro*

**FIGURE 6:**
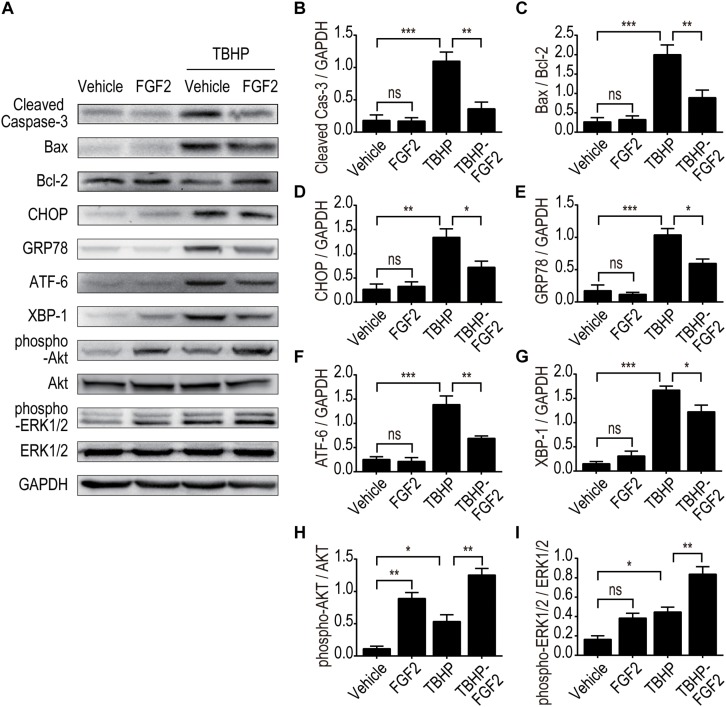
The effect of FGF2 on the inhibition of apoptosis and ER stress and the activation of the Akt and ERK1/2 signaling pathway was detected *in vitro*. **(A)** The expression of pro-apoptotic proteins, the expression of ER stress-relevant proteins, and the phosphorylation of Akt and ERK1/2 were detected by Western blotting. Images are representative of five animals in each group. **(B–I)** The histograms show the normalized optical density analysis. FGF2 significantly decreased the expression of cleaved Caspase-3, Bax, CHOP, GRP78, ATF-6, and XBP-1 in NRK-52E treated with TBHP. The phosphorylation of Akt and ERK1/2 was increased in the TBHP-FGF2 group compared with the TBHP group. Representative data of five individual samples in each group. *P < 0.05, **P < 0.01, ***P< 0.001; *ns* indicates not statistically significant.

To further clarify the role of FGF2 in the regulation of ER stress and the resistance to apoptosis, we determined the protective effect of FGF2 on NRK-52E cells induced by TBHP. The expressions of cleaved Caspase-3, Bax, and Bcl2 were detected by Western blotting, as described (Figure 6A). The results indicate that FGF2 treatment effectively reduced TBHP-induced cleaved Caspase-3 and Bax. Figures 6B,C the expression levels of CHOP, GRP78, ATF-6, and XBP-1 were also examined by Western blotting (Figure 6A). TBHP remarkably increased the expression of CHOP, GRP78, ATF-6, and XBP-1 in NRK-52E cells (Figures 6D–G). Similar to the results of the *in vivo* experiment, FGF2 significantly reduced the expression of ER stress-relevant proteins in NRK-52E cells treated with TBHP. Furthermore, the phosphorylation of AKT and ERK1/2 was significantly increased in the TBHP-FGF2 group compared to the TBHP group (Figures 6H,I). Different from the results of FGF2 treatment *in vivo*, FGF2 could active the Akt and ERK1/2 pathways in NRK-52E cells cultured with normal medium. There may be many reasons for the discrepancy, but the strong binding of FGF2 to heparan sulfate (HS) is likely one of the main causes. Many studies have reported that HS in the tissue can stabilize the biological functions of canonical paracrine/autocrine FGFs (Kan et al., 1988, 1991; Li et al., 2016). Tissue matrix HS acts as an FGF depot and limits the access of FGFs to cellular membrane FGFR except when needed (McKeehan et al., 1998).

**FIGURE 7:**
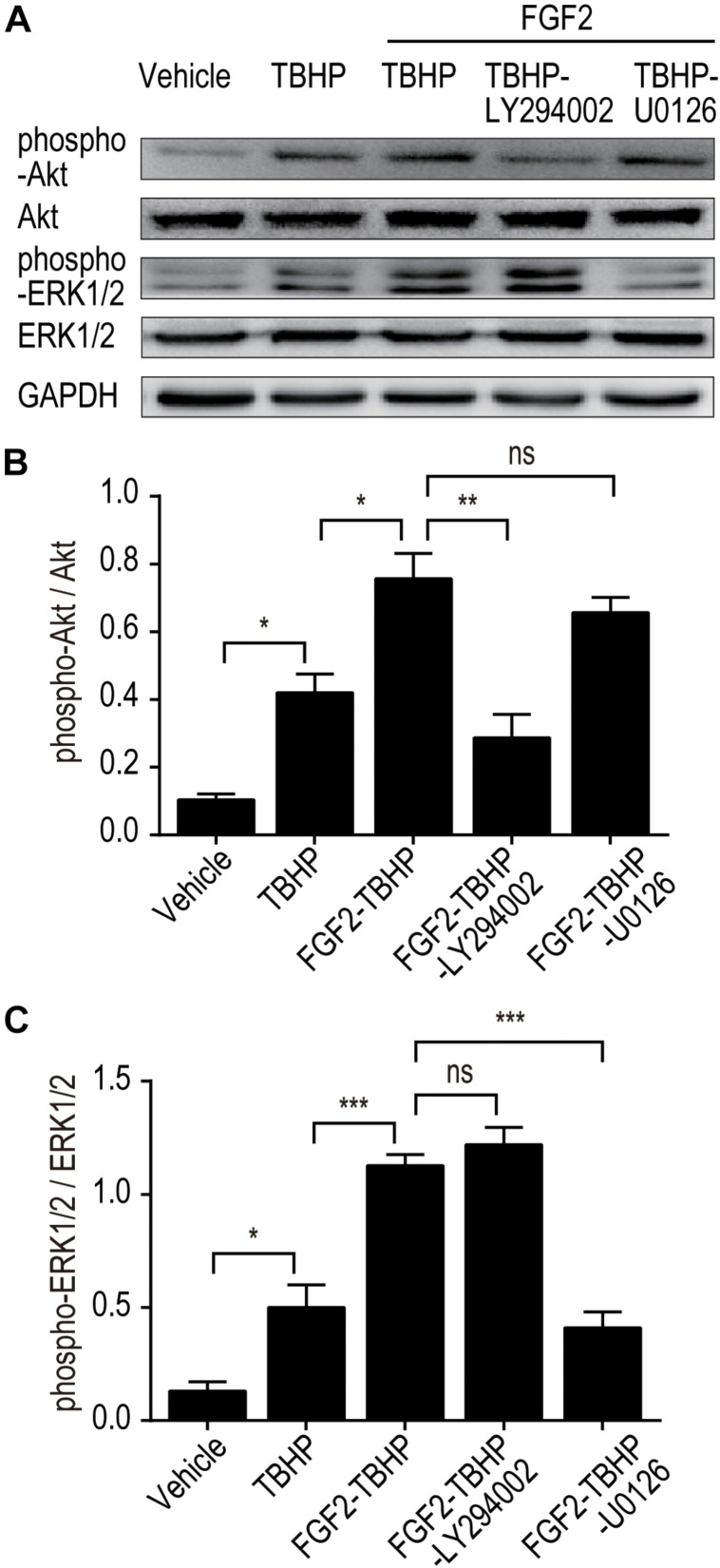
LY294002 and U0126 abolish the effect of FGF2 on the phosphorylation of Akt and ERK1/2 *in vitro*. **(A)** The phosphorylation of Akt and ERK1/2 was determined by Western blotting analysis. GAPDH was used as a loading control. Images are representative of five animals in each group. **(B,C)** The histograms show the normalized optical density analysis. LY294002 inhibited the effect of FGF2 treatment on the phosphorylation of Akt, whereas U0126 strikingly blocked the effect of FGF2 on the phosphorylation of ERK1/2. Representative data of five individual samples in each group. *P < 0.05, **P < 0.01, ***P< 0.001; *ns* indicates not statistically significant.

To further clarify whether PI3K/Akt or ERK1/2 pathways are involved in the protective effect of FGF2, we examined the phosphorylation of Akt and ERK1/2 *in vitro* using selective PI3K inhibitor (LY294002) and selective MEK inhibitor (U0126). As shown in Figure 7, FGF2 treatment significantly activated the phosphorylation of Akt and ERK1/2 compared to the TBHP group, which is consistent with the *in vivo* results. As expected, pre-addition of LY294002 and U0126 respectively largely abolished the effect of FGF2 on the activation of the PI3K/Akt and MEK-ERK1/2 signaling pathways in NRK-52E cells induced by TBHP.

**FIGURE 8:**
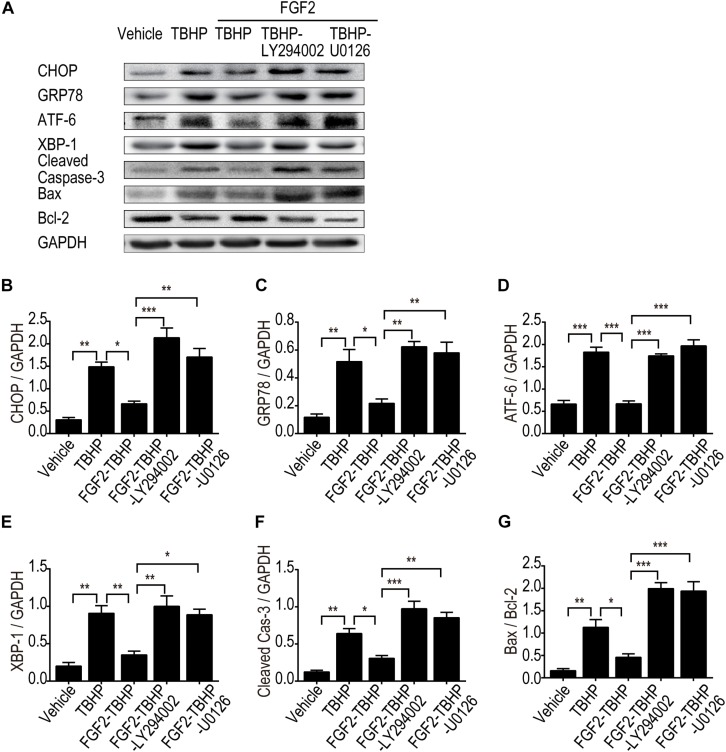
LY294002 and U0126 abolish the effect of FGF2 on the inhibition of ER stress and apoptosis *in vitro*. **(A)** The expression of ER stress-relevant proteins and pro-apoptotic proteins were detected by Western blotting. Images are representative of five animals in each group. **(B–G)** The histograms show the normalized optical density analysis. Both LY294002 and U0126 could largely suppress the effect of FGF2 on apoptosis and ER stress. Representative data of five individual samples in each group. *P < 0.05, **P < 0.01, ***P < 0.001.

To clarify the role of the PI3K/Akt and ERK1/2 pathways in the regulation of ER stress, the expression of ER stress-relevant proteins CHOP, GRP78, ATF-6, and XBP-1 was detected by Western blotting (Figures 8A–E). The results indicated that FGF2 significantly reduced the production of CHOP, GRP78, ATF-6, and XBP-1, whereas both LY294002 and U0126 inhibited the role of FGF2, individually. To clarify the role of the PI3K/Akt and ERK1/2 pathways in apoptosis, the expression of cleaved Caspase-3, Bax, and Bcl2 was detected by Western blotting (Figures 8A,F,G). The results indicated that FGF2 treatment effectively reduced the activation of Caspase-3 and the expression of Bax, whereas either LY294002 or U0126 abolished the protective effect of FGF2. These results strongly suggest that FGF2 could prevent excessive ER stress-mediated apoptosis in kidney tissues after I/R injury. The activation of the PI3K/Akt and MEK-ERK1/2 signaling pathways plays a crucial role in the protective effect of FGF2.

## Discussion

Acute kidney injury, mainly caused by renal I/R injury, remains a vexing health problem around the world. Despite the recent biological and clinical advances in supporting measures and prophylactic approaches, the mortality rate of AKI patients remains fairly high (Ishani et al., 2009; Winterberg and Lu, 2012). As a member of the FGF family, the role of FGF2 in organ development, tissue repair, regeneration, and diseases has been widely investigated (Beenken and Mohammadi, 2009). The protective effect of FGF2 has been investigated in several disease conditions, such as cerebral ischemia injury and wound healing (House et al., 2003; Wang et al., 2012, 2015; Zhang et al., 2013; Villanueva et al., 2014; Liu et al., 2018). In our previous study, we demonstrated that FGF2 could protect against renal I/R injury via attenuation of mitochondrial damage and HMGB1/TLR2-mediated inflammatory response (Tan et al., 2017). In addition, other findings revealed that ER stress is involved in AKI triggered by renal I/R injury and nephrotoxic drugs (Gallazzini and Pallet, 2018; Yan et al., 2018). Many studies have reported that ER stress could trigger inflammatory responses and regulate mitochondrial metabolic status through the UPR signaling pathway (Bronner et al., 2015; So, 2018; Gallazzini and Pallet, 2018; Inoue et al., 2019). However, whether ER stress is involved in the protective effect of FGF2 on AKI has not yet been fully clarified. Our present study provides *in vivo* and *in vitro* experimental evidence that exogenously administered FGF2 protects renal tubular epithelial cells against apoptosis via the regulation of ER stress. We also examined the role of the PI3K/Akt and ERK1/2 signaling pathways in the protective effect of FGF2.

Disturbances such as glucose depletion, hypoxia, and oxidative stress can lead to excessive ER stress and UPR, which have previously been demonstrated to perform a pivotal role in I/R-induced apoptosis and functional damage (Kim et al., 2008; Belaidi et al., 2013; Cao and Kaufman, 2014). The molecular mechanism underlying ER stress and UPR in renal I/R injury remains poorly understood. The association of ER stress and apoptosis in kidneys during AKI has also been reported (Inagi, 2009). Excessive ER stress for a prolonged period of time can elicit cell death and thus contributes to both glomerular and tubular damage in patients with AKI as well as chronic kidney diseases (Bhatt et al., 2008; Inagi, 2009; Xu et al., 2016). Many studies have reported that CHOP (C/EBP homologous proteins), also known as DDIT3 (DNA damage-inducible transcript 3), is the most important regulator of maladaptive ER stress-induced apoptosis (Rutkowski et al., 2006). Our results indicate that the expression of ER stress response proteins, including CHOP, GRP78, XBP-1, and ATF-6, was significantly increased upon I/R injury. Importantly, FGF2 treatment strikingly reduced the expression of relevant ER stress proteins after I/R injury. It is generally accepted that moderate ER stress is beneficial to cell survival, whereas excessive ER stress stimulates apoptotic pathways (Zhao and Ackerman, 2006). Our results suggest that the reno-protective effect of FGF2 against I/R injury in kidney is consequential to its alleviation of excessive ER stress.

The PI3K/Akt and MEK-ERK1/2 pathways are among the most commonly activated signaling pathways associated with various kidney diseases, including renal I/R injury (Tian et al., 2000; Zhang et al., 2018; Lieberthal et al., 2019). The PI3K/Akt and ERK1/2 signaling pathways are particularly important in mediating cell survival under a wide range of cellular circumstances. However, the molecular mechanisms of the PI3K/Akt and ERK1/2 signaling pathways underlying the protective effect of FGF2 on renal histology integrity and function remain poorly understood (Feliers and Kasinath, 2011; Zhang and Cai, 2016). To examine the potential role of the PI3K/Akt and ERK1/2 pathways in the protective effect of FGF2 on AKI, we examined the activation of PI3K/Akt and ERK1/2 in kidney tissues. Transient activations of the PI3K/Akt and MEK-ERK1/2 signaling pathways were detected in I/R kidneys at 6 h after reperfusion compared to in the Sham group. The increased expression of phosphor-Akt and phosphor-ERK1/2 indicate the self-activation of the PI3K/Akt and MEK-ERK1/2 signaling pathways in kidney tissues after reperfusion. It has been reported that both the PI3K/Akt and ERK1/2 signaling pathways can inhibit excessive ER stress after injury. Combined with the expression of relevant ER stress proteins in the I/R group, we inferred that the transient self-activation of the PI3K/Akt and ERK1/2 pathways was insufficient to inhibit the excessive ER stress. Moreover, FGF2 substantially improved the phosphorylation of Akt and ERK1/2 compared to the I/R group at 24 and 72 h after reperfusion. Based on these results, we concluded that the role of FGF2 in the regulation of ER stress after renal I/R injury is associated with the activation of the PI3K/Akt and ERK1/2 signaling pathways.

To further clarify the relationship between the PI3K/Akt and MEK-ERK1/2 pathways in the regulatory role of FGF2 on ER stress and apoptosis after reperfusion, we examined the protective effect of FGF2 in NRK-52E cells. Treatment of NRK-52E cells with TBHP, a widely used oxidative stress inducer, led to the activation of ER stress and apoptosis. In addition, blockade of PI3K/Akt and MEK-ERK1/2 signaling by LY294002 or U0126, respectively, can partially abrogate the anti-apoptotic activity of FGF2. Both LY294002 and U0126 can reverse the effect of FGF2 on the expression of ER stress-relevant proteins. Pretreatment of NRK-52E cells with LY294002 or U0126 also suppressed the phosphorylation of Akt or ERK1/2 and thus abolished the protective effect of FGF2 against apoptosis in renal tubular epithelial cells. Although our experimental results confirmed the role of the PI3K/Akt and MEK-ERK1/2 pathways in the protective effect of FGF2, the present study did not exclude the role of other signaling pathways that may be involved in the regulation of cells survival after I/R injury.

In summary, the present study demonstrated that FGF2 treatment attenuates renal tubular epithelial cell apoptosis via the inhibition of excessive ER stress. The regulatory role of FGF2 in ER stress and apoptosis is, at least partially, mediated by the activation of the PI3K/Akt and MEK-ERK1/2 signaling pathways. This study suggests that FGF2 treatment should be considered as a therapeutic strategy for acute kidney injury caused by I/R.

## Data Availability Statement

All datasets generated for this study are included in the article/supplementary material.

## Ethics Statement

The animal study was reviewed and approved by the Institutional Animal Ethical and Use Committee of Wenzhou Medical University.

## Author Contributions

XT and DL conceived and designed the experiments, and funded the study. XT and QT performed the animal operations. LX, GL, QT, XZ, and TZ performed apoptosis assay, immunoblot, immunohistochemistry, and immunofluorescence. XT and CW analyzed the data and prepared the figures. XT wrote and revised the manuscript.

## Conflict of Interest

The authors declare that the research was conducted in the absence of any commercial or financial relationships that could be construed as a potential conflict of interest.

## References

[B1] Basile D. P. Anderson M. D. Sutton T. A. (2012). Pathophysiology of acute kidney injury. *Compr. Physiol.* 2 1303–1353. 23798302 10.1002/cphy.c110041 PMC3919808

[B2] Beenken A. Mohammadi M. (2009). The FGF family: biology, pathophysiology and therapy. *Nat. Rev. Drug Discov.* 8 235–253. 10.1038/nrd2792 19247306 PMC3684054

[B3] Belaidi E. Decorps J. Augeul L. Durand A. Ovize M. (2013). Endoplasmic reticulum stress contributes to heart protection induced by cyclophilin D inhibition. *Basic Res. Cardiol.* 108:363. 10.1007/s00395-013-0363-z 23744057

[B4] Bhatt K. Feng L. P. Pabla N. Liu K. B. Smith S. Dong Z. (2008). Effects of targeted Bcl-2 expression in mitochondria or endoplasmic reticulum on renal tubular cell apoptosis. *Am. J. Physiol.-Renal.* 294 F499–F507. 10.1152/ajprenal.00415.2007 18160625

[B5] Bohle A. Jahnecke J. Meyer D. Schubert G. E. (1976). Morphology of acute renal failure: comparative data from biopsy and autopsy. *Kidney Int. Suppl.* 6 S9–S16. 1068332

[B6] Bonventre J. V. Yang L. (2011). Cellular pathophysiology of ischemic acute kidney injury. *J. Clin. Invest.* 121 4210–4221. 10.1172/jci45161 22045571 PMC3204829

[B7] Bright M. D. Clarke P. A. Workman P. Davies F. E. (2018). Oncogenic RAC1 and NRAS drive resistance to endoplasmic reticulum stress through MEK/ERK signalling. *Cell. Signal.* 44 127–137. 10.1016/j.cellsig.2018.01.004 29329780 PMC6562199

[B8] Brodsky J. L. Skach W. R. (2011). Protein folding and quality control in the endoplasmic reticulum: recent lessons from yeast and mammalian cell systems. *Curr. Opin. Cell Biol.* 23 464–475. 10.1016/j.ceb.2011.05.004 21664808 PMC3154734

[B9] Bronner D. N. Abuaita B. H. Chen X. Fitzgerald K. A. Nunez G. He Y. (2015). Endoplasmic reticulum stress activates the inflammasome via NLRP3- and caspase-2-driven mitochondrial damage. *Immunity* 43 451–462. 10.1016/j.immuni.2015.08.008 26341399 PMC4582788

[B10] Cai Z. Semenza G. L. (2004). Phosphatidylinositol-3-kinase signaling is required for erythropoietin-mediated acute protection against myocardial ischemia/reperfusion injury. *Circulation* 109 2050–2053. 10.1161/01.cir.0000127954.98131.23 15117842

[B11] Cao S. S. Kaufman R. J. (2014). Endoplasmic reticulum stress and oxidative stress in cell fate decision and human disease. *Antioxid. Redox. Signal.* 21 396–413. 10.1089/ars.2014.5851 24702237 PMC4076992

[B12] Chen H. Song Z. Ying S. Yang X. Wu W. Tan Q. (2018). Myeloid differentiation protein 2 induced retinal ischemia reperfusion injury via upregulation of ROS through a TLR4-NOX4 pathway. *Toxicol. Lett.* 282 109–120. 10.1016/j.toxlet.2017.10.018 29111459

[B13] Chertow G. M. Burdick E. Honour M. Bonventre J. V. Bates D. W. (2005). Acute kidney injury, mortality, length of stay, and costs in hospitalized patients. *J. Am. Soc. Nephrol.* 16 3365–3370. 10.1681/asn.2004090740 16177006

[B14] Cybulsky A. V. (2017). Endoplasmic reticulum stress, the unfolded protein response and autophagy in kidney diseases. *Nat. Rev. Nephrol.* 13 681–696. 10.1038/nrneph.2017.129 28970584

[B15] di Mari J. F. Davis R. Safirstein R. L. (1999). MAPK activation determines renal epithelial cell survival during oxidative injury. *Am. J. Physiol.* 277 F195–F203. 10444573 10.1152/ajprenal.1999.277.2.F195

[B16] Eltzschig H. K. Eckle T. (2011). Ischemia and reperfusion–from mechanism to translation. *Nat. Med.* 17 1391–1401. 10.1038/nm.2507 22064429 PMC3886192

[B17] Feliers D. Kasinath B. S. (2011). Erk in kidney diseases. *J. Signal. Transduct.* 2011:768512. 10.1155/2011/768512 PMC3135240 21776388

[B18] Fernandes-Alnemri T. Litwack G. Alnemri E. S. (1994). CPP32, a novel human apoptotic protein with homology to Caenorhabditis elegans cell death protein Ced-3 and mammalian interleukin-1 beta-converting enzyme. *J. Biol. Chem.* 269 30761–30764. 7983002

[B19] Gallazzini M. Pallet N. (2018). Endoplasmic reticulum stress and kidney dysfunction. *Biol. Cell* 110 205–216. 10.1111/boc.201800019 29989181

[B20] Guo F. J. Liu Y. Zhou J. Luo S. Zhao W. Li X. (2012). XBP1S protects cells from ER stress-induced apoptosis through Erk1/2 signaling pathway involving CHOP. *Histochem. Cell Biol.* 138 447–460. 10.1007/s00418-012-0967-7 22669460

[B21] Hetz C. (2012). The unfolded protein response: controlling cell fate decisions under ER stress and beyond. *Nat. Rev. Mol. Cell Biol.* 13 89–102. 10.1038/nrm3270 22251901

[B22] House S. L. Bolte C. Zhou M. Doetschman T. Klevitsky R. Newman G. (2003). Cardiac-specific overexpression of fibroblast growth factor-2 protects against myocardial dysfunction and infarction in a murine model of low-flow ischemia. *Circulation* 108 3140–3148. 10.1161/01.cir.0000105723.91637.1c 14656920

[B23] Hsu H. S. Liu C. C. Lin J. H. Hsu T. W. Hsu J. W. Su K. (2017). Involvement of ER stress, PI3K/AKT activation, and lung fibroblast proliferation in bleomycin-induced pulmonary fibrosis. *Sci. Rep.* 7:14272. 10.1038/s41598-017-14612-5 PMC5660192 29079731

[B24] Inagi R. (2009). Endoplasmic reticulum stress in the kidney as a novel mediator of kidney injury. *Nephron Exp. Nephrol.* 112 E1–E9. 19342868 10.1159/000210573

[B25] Inoue T. Maekawa H. Inagi R. (2019). Organelle crosstalk in the kidney. *Kid. Int.* 95 1318–1325. 10.1016/j.kint.2018.11.035 30878214

[B26] Ishani A. Xue J. L. Himmelfarb J. Eggers P. W. Kimmel P. L. Molitoris B. A. (2009). Acute kidney injury increases risk of ESRD among elderly. *J. Am. Soc. Nephrol.* 20 223–228. 10.1681/asn.2007080837 19020007 PMC2615732

[B27] Kan M. DiSorbo D. Hou J. Z. Hoshi H. Mansson P. E. McKeehan W. L. (1988). High and low affinity binding of heparin-binding growth factor to a 130-kDa receptor correlates with stimulation and inhibition of growth of a differentiated human hepatoma cell. *J. Biol. Chem.* 263 11306–11313. 2457020

[B28] Kan M. Shi E. G. McKeehan W. L. (1991). Identification and assay of fibroblast growth factor receptors. *Methods Enzymol.* 198 158–171. 10.1016/0076-6879(91)98017-z 1713285

[B29] Kim I. Xu W. Reed J. C. (2008). Cell death and endoplasmic reticulum stress: disease relevance and therapeutic opportunities. *Nat. Rev. Drug Discov.* 7 1013–1030. 10.1038/nrd2755 19043451

[B30] Kim S. Woo C. H. (2018). Laminar flow inhibits er stress-induced endothelial apoptosis through PI3K/Akt-dependent signaling pathway. *Mol. Cells* 41 964–970. 30396238 10.14348/molcells.2018.0111 PMC6277562

[B31] Kwon D. S. Kwon C. H. Kim J. H. Woo J. S. Jung J. S. Kim Y. K. (2006). Signal transduction of MEK/ERK and PI3K/Akt activation by hypoxia/reoxygenation in renal epithelial cells. *Eur. J. Cell Biol.* 85 1189–1199. 10.1016/j.ejcb.2006.06.001 16860436

[B32] Li X. Wang C. Xiao J. McKeehan W. L. Wang F. (2016). Fibroblast growth factors, old kids on the new block. *Semin. Cell Dev. Biol.* 53 155–167. 10.1016/j.semcdb.2015.12.014 26768548 PMC4875805

[B33] Lieberthal W. Tang M. Abate M. Lusco M. Levine J. S. (2019). AMPK-mediated activation of Akt protects renal tubular cells from stress-induced apoptosis in vitro and ameliorates ischemic AKI in vivo. *Am. J. Physiol. Renal Physiol.* 317 F1–F11. 30995114 10.1152/ajprenal.00553.2018

[B34] Linkermann A. Chen G. Dong G. Kunzendorf U. Krautwald S. Dong Z. (2014). Regulated cell death in AKI. *J. Am. Soc. Nephrol.* 25 2689–2701. 10.1681/asn.2014030262 24925726 PMC4243360

[B35] Liu M. Wu Y. Liu Y. Chen Z. He S. Zhang H. (2018). Basic fibroblast growth factor protects astrocytes against ischemia/reperfusion injury by upregulating the caveolin-1/VEGF signaling pathway. *J. Mol. Neurosci.* 64 211–223. 10.1007/s12031-017-1023-9 29299743

[B36] McKeehan W. L. Wang F. Kan M. (1998). The heparan sulfate-fibroblast growth factor family: diversity of structure and function. *Prog. Nucleic Acid Res. Mol. Biol.* 59 135–176. 10.1016/s0079-6603(08)61031-4 9427842

[B37] Paller M. S. Manivel J. C. (1992). Prostaglandins protect kidneys against ischemic and toxic injury by a cellular effect. *Kid. Int.* 42 1345–1354. 10.1038/ki.1992.426 1474766

[B38] Pearson G. Robinson F. Beers Gibson T. Xu B. E. Karandikar M. Berman K. (2001). Mitogen-activated protein (MAP) kinase pathways: regulation and physiological functions. *Endocr. Rev.* 22 153–183. 10.1210/er.22.2.153 11294822

[B39] Rutkowski D. T. Arnold S. M. Miller C. N. Wu J. Li J. Gunnison K. M. (2006). Adaptation to ER stress is mediated by differential stabilities of pro-survival and pro-apoptotic mRNAs and proteins. *PLoS Biol.* 4:e374. 10.1371/journal.pbio.0040374 17090218 PMC1634883

[B40] Schumer M. Colombel M. C. Sawczuk I. S. Gobe G. Connor J. O’Toole K. M. (1992). Morphologic, biochemical, and molecular evidence of apoptosis during the reperfusion phase after brief periods of renal ischemia. *Am. J. Pathol.* 140 831–838. 1562048 PMC1886381

[B41] Shen D. Chen R. Zhang L. Rao Z. Ruan Y. Li L. (2019). Sulodexide attenuates endoplasmic reticulum stress induced by myocardial ischaemia/reperfusion by activating the PI3K/Akt pathway. *J. Cell Mol. Med.* 23 5063–5075. 10.1111/jcmm.14367 31120192 PMC6653332

[B42] So J. S. (2018). Roles of endoplasmic reticulum stress in immune responses. *Mol. Cells* 41 705–716. 30078231 10.14348/molcells.2018.0241 PMC6125421

[B43] Soares R. O. S. Losada D. M. Jordani M. C. Evora P. Castro E. S. O. (2019). Ischemia/reperfusion injury revisited: an overview of the latest pharmacological strategies. *Int. J. Mol. Sci.* 20:E5034. 10.3390/ijms20205034 PMC6834141 31614478

[B44] Tan X. Zhang L. Jiang Y. Yang Y. Zhang W. Li Y. (2013). Postconditioning ameliorates mitochondrial DNA damage and deletion after renal ischemic injury. *Nephrol. Dial. Transplant.* 28 2754–2765. 10.1093/ndt/gft278 24021677 PMC3811057

[B45] Tan X. H. Zheng X. M. Yu L. X. He J. Zhu H. M. Ge X. P. (2017). Fibroblast growth factor 2 protects against renal ischaemia/reperfusion injury by attenuating mitochondrial damage and proinflammatory signalling. *J. Cell. Mol. Med.* 21 2909–2925. 10.1111/jcmm.13203 28544332 PMC5661260

[B46] Tian W. Zhang Z. Cohen D. M. (2000). MAPK signaling and the kidney. *Am. J. Physiol. Renal Physiol.* 279 F593–F604. 10997909 10.1152/ajprenal.2000.279.4.F593

[B47] Villanueva S. Cespedes C. Vio C. P. (2006). Ischemic acute renal failure induces the expression of a wide range of nephrogenic proteins. *Am. J. Physiol. Regul. Integr. Compar. Physiol.* 290 R861–R870. 10.1152/ajpregu.00384.2005 16284088

[B48] Villanueva S. Contreras F. Tapia A. Carreno J. E. Vergara C. Ewertz E. (2014). Basic fibroblast growth factor reduces functional and structural damage in chronic kidney disease. *Am. J. Physiol. Renal Physiol.* 306 F430–F441. 24285501 10.1152/ajprenal.00720.2012

[B49] Walter P. Ron D. (2011). The unfolded protein response: from stress pathway to homeostatic regulation. *Science* 334 1081–1086. 10.1126/science.1209038 22116877

[B50] Wang Z. Zhang H. Xu X. Shi H. Yu X. Wang X. (2012). bFGF inhibits ER stress induced by ischemic oxidative injury via activation of the PI3K/Akt and ERK1/2 pathways. *Toxicol. Lett.* 212 137–146. 10.1016/j.toxlet.2012.05.006 22609091

[B51] Wang Z. G. Wang Y. Ye J. M. Lu X. H. Cheng Y. Xiang L. J. (2015). bFGF attenuates endoplasmic reticulum stress and mitochondrial injury on myocardial ischaemia/reperfusion via activation of PI3K/Akt/ERK1/2 pathway. *J. Cell Mol. Med.* 19 595–607. 10.1111/jcmm.12346 25533999 PMC4369816

[B52] Winterberg P. D. Lu C. Y. (2012). Acute kidney injury: the beginning of the end of the dark ages. *Am. J. Med. Sci.* 344 318–325. 10.1097/maj.0b013e318228aef8 21817881 PMC3210873

[B53] Wu M. Y. Yiang G. T. Liao W. T. Tsai A. P. Cheng Y. L. Cheng P. W. (2018). Current mechanistic concepts in ischemia and reperfusion injury. *Cell Physiol. Biochem.* 46 1650–1667. 29694958 10.1159/000489241

[B54] Wu S. Z. Tao L. Y. Wang J. N. Xu Z. Q. Wang J. Xue Y. J. (2017). Amifostine pretreatment attenuates myocardial ischemia/reperfusion injury by inhibiting apoptosis and oxidative stress. *Oxid. Med. Cell Longev.* 2017:4130824. 10.1155/2017/4130824 PMC5368387 28392886

[B55] Xiao J. Lv Y. Lin S. Jin L. Zhang Y. Wang X. (2010). Cardiac protection by basic fibroblast growth factor from ischemia/reperfusion-induced injury in diabetic rats. *Biol. Pharm. Bull.* 33 444–449. 10.1248/bpb.33.444 20190407

[B56] Xu Y. Guo M. Jiang W. Dong H. Han Y. F. An X. F. (2016). Endoplasmic reticulum stress and its effects on renal tubular cells apoptosis in ischemic acute kidney injury. *Ren Fail* 38 831–837. 10.3109/0886022x.2016.1160724 27001462

[B57] Yan M. Shu S. Guo C. Tang C. Dong Z. (2018). Endoplasmic reticulum stress in ischemic and nephrotoxic acute kidney injury. *Ann. Med.* 50 381–390. 10.1080/07853890.2018.1489142 29895209 PMC6333465

[B58] Zhang G. Wang Q. Wang W. Yu M. Zhang S. Xu N. (2018). Tempol protects against acute renal injury by regulating PI3K/Akt/mTOR and GSK3beta signaling cascades and afferent arteriolar activity. *Kid. Blood Press. Res.* 43 904–913. 10.1159/000490338 29870982 PMC6065105

[B59] Zhang H. Y. Zhang X. Wang Z. G. Shi H. X. Wu F. Z. Lin B. B. (2013). Exogenous basic fibroblast growth factor inhibits ER stress-induced apoptosis and improves recovery from spinal cord injury. *CNS Neurosci. Ther.* 19 20–29. 10.1111/cns.12013 23082997 PMC6493620

[B60] Zhang Z. Cai C. X. (2016). Kidney injury molecule-1 (KIM-1) mediates renal epithelial cell repair via ERK MAPK signaling pathway. *Mol. Cell. Biochem.* 416 109–116. 10.1007/s11010-016-2700-7 27084535 PMC4883006

[B61] Zhao L. Ackerman S. L. (2006). Endoplasmic reticulum stress in health and disease. *Curr. Opin. Cell. Biol.* 18 444–452. 16781856 10.1016/j.ceb.2006.06.005

